# Differential Contributions of IgM and IgG Autoantibodies to Serologic IA2 Reactivity in Type 1 Diabetes

**DOI:** 10.3390/biom16040500

**Published:** 2026-03-26

**Authors:** Xuming Mao, Jake Konigsberg, Nadia Noorchashm, Wenzhao Meng, James J. Knox, Gregory J. Golden, Jacob T. Hamilton, Tara K. Maxwell, Chengyang Liu, Michael R. Betts, Steven M. Willi, Ali Naji, Patrick Hanley, Eline T. Luning Prak

**Affiliations:** 1 Department of Pathology and Laboratory Medicine, Perelman School of Medicine, University of Pennsylvania, Philadelphia, PA 19104, USA; maox@pennmedicine.upenn.edu (X.M.); jakekon@sas.upenn.edu (J.K.); nadia.f.noorchashm@pennmedicine.upenn.edu (N.N.); wmeng@pennmedicine.upenn.edu (W.M.); knoxjam@pennmedicine.upenn.edu (J.J.K.); 2 Breakthrough T1D, New York, NY 10281, USA; ggolden@breakthrought1d.org; 3 Institute for Immunology, Perelman School of Medicine, University of Pennsylvania, Philadelphia, PA 19104, USA; jacob.hamilton@pennmedicine.upenn.edu (J.T.H.); betts@pennmedicine.upenn.edu (M.R.B.); ali.naji@pennmedicine.upenn.edu (A.N.); 4 Department of Microbiology, Perelman School of Medicine, University of Pennsylvania, Philadelphia, PA 19104, USA; 5 Division of Endocrinology and Diabetes, Children’s Hospital of Philadelphia, Philadelphia, PA 19104, USA; maxwellt@chop.edu (T.K.M.); willi@chop.edu (S.M.W.); 6 Department of Surgery, Perelman School of Medicine, University of Pennsylvania, Philadelphia, PA 19104, USA; chliu@pennmedicine.upenn.edu; 7 Colton Center for Autoimmunity, Perelman School of Medicine, University of Pennsylvania, Philadelphia, PA 19104, USA; 8 Division of Endocrinology, Nemours Children’s Hospital, Wilmington, DE 19803, USA; patrick.hanley@nemours.org

**Keywords:** type 1 diabetes (T1D), autoantibodies, islet antigen 2 (IA2), Immunoglobulin G (IgG), Immunoglobulin M (IgM), electrochemiluminescence (ECL)

## Abstract

Autoantibodies targeting islet antigen 2 (IA2) are critical diagnostic and prognostic markers for type 1 diabetes (T1D). Standard clinical assays do not differentiate between IgG and IgM isotypes, yet these antibodies have distinct roles in the T1D autoimmunity. We therefore adapted electrochemiluminescence (ECL) assays to separately detect IgG and IgM antibodies against the IA2 intracellular domain (AA601-979). Assay specificity was confirmed by indirect immunofluorescence, which showed autoantibody binding to IA2-overexpressing cells. Plasma samples were analyzed from two independent cohorts: organ donors of the Human Pancreas Analysis Program (HPAP, *n* = 69) and children from a Janssen–Breakthrough T1D-funded study (*n* = 65). Diabetics had significantly higher levels of IA2 IgG (*p* < 0.001) but not IgM (*p* > 0.05) compared with controls. Notably, IgM and IgG IA2 antibody levels were not correlated. However, IgM modulates IgG detection: IgM depletion increased detected IgG levels to IA2 in some donors, and sera from donors with high IA2-specific IgM levels reduced monoclonal IgG anti-IA2 antibody binding to IA2. Purified IgM from healthy individuals also suppressed monoclonal IgG binding. These findings support distinct, non-redundant roles for IA2-specific IgG and IgM in T1D serology. Isotype-specific autoantibody analysis may improve risk stratification and monitoring of T1D individuals receiving immunomodulatory therapies.

## 1. Introduction

Type 1 diabetes (T1D) is a chronic autoimmune disease characterized by immune-mediated destruction of insulin-producing β-cells within the pancreatic islets [1,2]. This debilitating condition arises from a complex interplay of genetic susceptibility, environmental factors, and immunological dysregulation, ultimately leading to autoimmunity against β-cells [3,4]. Circulating autoantibodies can emerge years before clinical hyperglycemia and serve as crucial biomarkers for risk prediction and disease monitoring [2,5,6,7]. Known autoantibodies target key β-cell antigens, including insulin (IAA) [8,9,10,11], glutamic acid decarboxylase 65 kD isoform (GAD65) [11,12], islet antigen-2 (IA2, insulinoma-associated antigen) [13,14], and zinc transporter 8 (ZnT8) [15].

Autoantibodies to islet IA2, a protein tyrosine phosphatase-like molecule expressed in β-cells, are particularly significant for predicting disease development [6,14,16]. IA2 autoantibodies are found in 50–80% of newly diagnosed T1D patients, with prevalence varying depending on assay methodology [17,18]. IA2 autoantibodies typically emerge in later stages of T1D progression [19,20] and are more frequently observed in older children and adolescents at diagnosis [21]. Their presence is associated with an elevated risk of rapid disease progression [7] and, when combined with other autoantibodies, their detection enhances diagnostic sensitivity for T1D [22]. Moreover, IA2 autoantibodies exhibit high specificity for T1D, aiding in differentiation from other forms of diabetes [21,23]. Autoantibody status, glycemic status, and clinical presentation define stages of disease progression [24].

A gap persists in our knowledge of the distinct contributions of IgM and IgG autoantibody isotypes to human T1D, both in terms of risk assessment and disease mechanisms. IgG and IgM antibodies not only reflect distinct phases of an immune response but may contribute to disease pathogenesis through distinct Fc receptor-mediated mechanisms and complement activation [25,26]. IgM, traditionally considered a first-line humoral response to foreign antigens, can also exhibit autoreactive specificity. IgG autoantibodies suggest the development of a T cell-dependent, affinity-matured memory response, with somatic hypermutation contributing to the potential development of autoreactive specificities [7,27,28]. Hence, the presence of IgG antibodies is more likely to reflect an established autoimmune response than the presence of only IgM autoantibodies.

While abnormal levels of total IgG and IgM have been reported in T1D [29,30], isotype-specific autoantibody assays are not routinely used for clinical evaluation. Current clinical assays are largely isotype-agnostic, either detecting all autoantibody classes and reporting a global “antibody index,” or focusing exclusively on IgG. Furthermore, standardization challenges across clinical laboratories have necessitated centralized testing [30,31], often relying on the historically established radiobinding assay (RBA) as the “gold standard” assay. The RBA suffers from drawbacks, including high cost, radioactivity, and limited throughput. Electrochemiluminescence (ECL)-based assays have emerged as promising non-radioactive alternatives, exhibiting improved predictive values and multiplexing capabilities [31,32,33]. However, currently employed bridging ECL assays are predominantly isotype-agnostic.

To address the need for isotype-specific autoantibody analysis, we developed ECL-based assays to separately quantify autoreactive IgG and IgM against the intracellular domain of IA2. We evaluated the ECL assays by comparing them with an RBA, a bridging assay, and a cell-based assay with transfected IA2. Next, we used the isotype-specific ECL assays to analyze circulating autoantibodies in two independent cohorts of individuals with and without T1D. We show that IgG, but not IgM, IA2 autoantibodies are associated with T1D and that IgG and IgM antibodies do not correlate with each other. We also show that IgM anti-IA2 antibodies from T1D and normal individuals can inhibit IgG binding to IA2. These findings highlight non-redundant contributions of IgG and IgM autoantibodies to T1D evaluation. We hypothesize that dissecting the isotype-specific responses to IA2 would provide new insights into the autoantibody response of T1D and potentially refine diagnostic assays and predictive strategies.

## 2. Materials and Methods

### 2.1. Subjects

Serum samples were randomly selected from two cohorts: the Human Pancreas Analysis Program (HPAP) organ donors (*n* = 69) and paired kindreds (children with and without T1D in the same family; *n* = 65 total) from a Janssen–Breakthrough T1D (formerly JDRF)-funded study (hereafter referred to as Breakthrough T1D, Table 1). The HPAP is an NIH-funded study in which investigators are performing deep phenotyping and multi-omics profiling of the pancreas and the immune system in human organ donors [34]. HPAP samples included plasma from control donors, donors who tested positive for at least one of four autoantibodies (IAA, GAD65, IA2, and ZnT8; AAb+), and donors who had been diagnosed with T1D (Supplementary Table S1). Autoantibody testing of the HPAP donors was performed in the Autoantibody/HLA Core Facility at the University of Colorado Anschutz Medical Campus, and data for each donor are provided in the HPAP data portal, PANC-DB (https://hpap.pmacs.upenn.edu/, URL accessed on 12 January 2025). Breakthrough T1D samples were collected from children diagnosed with T1D and their siblings (Supplementary Table S2).

### 2.2. Monoclonal Antibodies

FACS was performed on splenocytes to obtain IA2-specific B cells for monoclonal antibody production. Protein probes were created by conjugating recombinant human IA2 (PTPRN-01H; Creative BioMart, Shirley, NY, USA) with AF488 or AF647, using lightning-link kits (332-0030 and 336-0030; Novus Biologicals, Centennial, CO, USA) and manufacturer directions. B cells were identified as CD19^+^CD3^−^CD14^−^CD16^−^ live singlets, and IA2-specific B cells were identified by the simultaneous binding of both AF488- and AF647-conjugated probes. The antibody sequences of the variable domains from paired heavy and light chains were obtained through single-cell sequencing data (Chromium v2 BCR sequencing; 10× Genomics, Pleasanton, CA, USA) from HPAP donors diagnosed with T1D (J. Knox and E. Luning Prak, manuscript in preparation). IgH/IgL sequences were inserted into a human IgG1 backbone and expressed in 293HEK cells (Sino Biological, Houston, TX, USA). Purified monoclonal antibodies (HPAP mAb#16 and mAb#19 from Sino Biological) were visualized on 4–20% SDS-PAGE gel electrophoresis. Binding to the autoantigen, IA2, was confirmed using ECL assays.

### 2.3. ELISA

ELISAs were performed on high-binding 96-well plates (9018; Corning Inc., Corning NY, USA). Wells were coated with 100 µL of 4 µg/mL IA2 protein diluted in carbonate-bicarbonate buffer (C3041; Millipore Sigma, Burlington, MA, USA) and incubated overnight at 4 °C. After washing, wells were blocked with 2% BSA (A7906; Millipore Sigma) in phosphate-buffered saline (PBS) for 1 h at room temperature (RT). Samples, controls, and blanks were added to the wells (1:20 dilution in 100 µL 2% BSA in PBS) and incubated for 1 h at RT. HRP-conjugated detection antibody (b6759; Abcam, Cambridge, UK) was then added (1:2000 dilution, 100 µL 2% BSA in PBS) and incubated for 1 h at RT. Between steps, wells were washed 3× with PBS-T (PBS with 0.05% Tween). Signal development was achieved using 100 µL of TMB substrate (N301; Thermo Fisher, Waltham, MA, USA), incubating in the dark for 15 min, followed by termination with 50 µL of 1 M H_2_SO_4_. Absorbance was read at 450 nm using a microplate reader (ClarioStar; BMG LabTech, Cary, NC, USA).

### 2.4. Labeling of IA2 with Biotin and Sulfo-Tag

Recombinant IA2 protein (specifically the intracellular portion, hereafter termed “IA2”, AA601-979) was obtained from Surmodics (A305; Eden Prairie, MN, USA), diluted to 1 µg/µL in PBS, and biotinylated using a kit from Abcam (ab201795). For sulfo-tagging, 1 µg/µL of IA2 was mixed with the sulfo-tag reagents (R31AA1; Meso Scale Discovery, Rockville, MD, USA; hereafter “MSD”) at a molar ratio of 1:15, following manufacturer instructions. This mixture was incubated in the dark at RT for 2 h, then passed through a size-exclusion spin column (89808; Thermo Fisher) that had been equilibrated with conjugation buffer (R31AA1; MSD).

### 2.5. IA2 Autoantibody IgG and IgM Detection Using ECL

A total volume of 30 µL of diluted IA2 protein (2 µg/mL) was coated onto MSD GOLD 96-well small-spot streptavidin sector plates (L45SA5; MSD). Reactivity of IA2 IgG and IgM was evaluated using an MSD ECL, as illustrated in Supplementary Figure S1. After coating with antigen, plates were washed with wash buffer (PBST, 0.05% TWEEN PBS). Then, 30 µL of diluted plasma (1:50 in PBS) was added to each well and incubated with shaking at 700 rpm for 1 h at RT. Binding of IgG or IgM was detected using anti-human IgG (D21ADF-3; MSD) or IgM (D20JP-6; MSD) conjugated with SULFO-TAGs. After washing with PBST, a reading buffer (R92TG-2; MSD) was added to the plates, and the plates were read using the default settings (Meso QuickPlex SQ 1200 mm; MSD). Healthy human plasma, a monoclonal IA2 antibody (0.25 µg/mL), and/or a confirmed positive sample (1:50 dilution) from the HPAP cohort were used as controls.

### 2.6. IA2 Autoantibody Bridging Test Using ECL

The bridging ECL assay is shown in Supplementary Figure S1. Acid treatment of plasma followed a previously established protocol [30]. Briefly, 15 µL of each plasma sample was mixed with 18 µL of 0.5 mol/L acetate (035566.K7; Thermo Fisher). After incubation at RT for 45 min, 25 µL of each treated sample was transferred to a fresh tube containing 8.3 µL of neutralization buffer (1 M Tris-HCl, pH 9.0) and 35 µL of IA2 solution (which had been biotinylated and sulfo-tagged). The binding mixture was incubated at RT for 2 h, followed by an overnight at 4 °C with shaking (200 rpm). MSD GOLD 96-well small-spot streptavidin sector plates (L45SA-2; MSD) were blocked with 50 µL 3% BSA blocker buffer at 4 °C overnight. After washing with PBST, 30 µL of the binding mixture was added to the pre-blocked plates and incubated at RT for 1 h. The plates were washed three times with PBST, after which 150 µL of reading buffer (R92TG-2; MSD) was added to each well. Results were acquired using the Meso QuickPlex SQ 1200 mm.

### 2.7. Depletion of IgM from Plasma Samples

To deplete IgM from plasma, 1 µg of biotinylated anti-human IgM antibody (314504; BioLegend, San Diego, CA, USA) was added to plasma samples diluted 1:50 in PBS. The mixture was incubated with shaking at 200 rpm at RT for 1 h. Next, 15 µL of Streptavidin Dynabeads (60801D; Invitrogen, Carlsbad, CA, USA) were added to each sample, and incubation continued for an additional 1 h with shaking at 200 rpm at RT. The IgM-bound Dynabeads were then magnetically separated from the liquid sample, and the IgM-depleted plasma supernatants were collected. Both the supernatants and Dynabead fractions were retained for further analysis.

### 2.8. IgM Inhibition Experiment

To assess the competition or inhibitory effect of autoreactive IgM, 2 µL of plasma samples (which contained high levels of IgM anti-IA2 antibodies but low levels of IgG anti-IA2 antibodies) or 10 µL of purified IgM beads were added to diluted plasma samples. The mixtures were incubated with shaking at 200 rpm at RT for 1 h. After incubation, the plasma supernatants were collected for IgG IA2 binding assays using ECL.

### 2.9. Expression and Detection of IA2 with Indirect Immunofluorescence in Cultured Cells

C-terminal (amino acids 601–979) fragments of IA-2 were synthesized with codon optimization (Genscript, Piscataway, NJ, USA) and engineered to include a C-terminal 6× His tag. Each construct was subcloned separately into the mammalian expression vector pcDNA3.4 (Genscript). The resulting plasmids were used to transfect HaCat cells, a human epidermal cell line. At 24 h post-transfection, IA-2 protein expression was validated by staining with an anti-His antibody. Cells were subsequently fixed with 4% paraformaldehyde in PBS (J61899.AK; Thermo Fisher) and permeabilized with 0.25% Triton X-100 in PBS, blocked with 2% bovine serum albumin (BSA), and incubated overnight at 4 °C with human sera diluted 1:50 in 2% BSA. Bound serum antibodies were visualized using an AF488-conjugated secondary antibody (A-11001; Thermo Fisher). Nuclei were counterstained with DAPI using ProLong™ Gold Antifade Mounting with DAPI (P36941; Invitrogen). Fluorescence images were acquired with a Leica DM6000 confocal microscope (Leica Microsystems, Buffalo Grove, IL, USA) under identical acquisition settings for all samples.

### 2.10. Statistical Analysis

Statistical analyses and figure generation were performed using GraphPad Prism (v10), Past (v4.17), and Python (v3.11.7). Comparison of the ECL bridging assay to the IA2 antibody index was performed using linear regression and Spearman correlation analysis. A comparison of IA2 Autoantibodies (IgG and IgM) among the control, AAb+, and T1D donors was conducted using the Mann–Whitney or Kruskal–Wallis tests. Dunn’s Test was used for post hoc test corrections. Distribution plots of IgG and IgM levels were generated using a custom Python script employing the Seaborn (v0.13.2) visualization package. The Spearman test was used to test linear correlation. The correlation of IA2 IgG or IgM reactivities with demographic factors and autoantibody indices from the reference laboratory was assessed using Kendall’s Tau test. Bonferroni Correction was used for post hoc test corrections. The Wilcoxon signed-rank test was used for paired comparisons of IgG and IgM levels between patients with T1D and their first-degree relatives. A two-tailed *p*-value of less than 0.05 was considered statistically significant. Receiver Operating Characteristic analysis (ROC) of ECL IgG and IgM assays was conducted to evaluate the diagnostic performance of ECL IgG and IgM using GraphPad Prism (v10).

## 3. Results

### 3.1. Comparison of In-House IA2 Autoantibody ECL Bridging Assay to Other Assays

The goal of this study is to measure IA2-specific autoantibodies in plasma samples from two independent study cohorts using the ECL assay (Table 1). To validate our in-house ECL assay platform, we compared the levels of IA2 autoantibodies in plasma samples from our ECL bridging assay (see Methods) with the IA2 antibody index previously determined by the reference laboratory at the University of Colorado, as previously described [30,31]. Our results showed a strong correlation with the externally measured IA2 autoantibody index in the same 21 samples, with an R^2^ of 0.87 and a *p*-value of <0.05 (Supplementary Figure S2). A significant correlation was observed using the Spearman correlation analysis (r = 0.9741, *p* < 0.0001). Next, we compared our ECL assay with an ELISA using the same antigen source and a monoclonal anti-IA2 antibody (HPAP mAb#19), which was titrated in plasma from an antibody-negative individual. In this analysis, the ELISA showed lower sensitivity and a more limited dynamic range than the ECL assay (Supplementary Figure S3).

### 3.2. Parallel Evaluation of IgG and IgM Binding to IA2 in HPAP Samples Using Singleplex ECL Assays

To evaluate our IgM and IgG anti-IA2 ECL assays, we analyzed samples from two study cohorts. The first cohort consisted of 69 human organ donors from the NIH-funded Human Pancreas Analysis Program (HPAP, [34]) (Table 1). These extensively studied HPAP donors exhibit a range of autoantibody levels and disease phenotypes and have been classified as control, autoantibody-positive (AAb+), or T1D, with publicly available data [34]. The second cohort, recruited using funding from Breakthrough T1D (formerly JDRF) and Janssen, consisted of 65 children and their siblings, with and without T1D, based on clinical criteria (Table 1).

**Figure 1:**
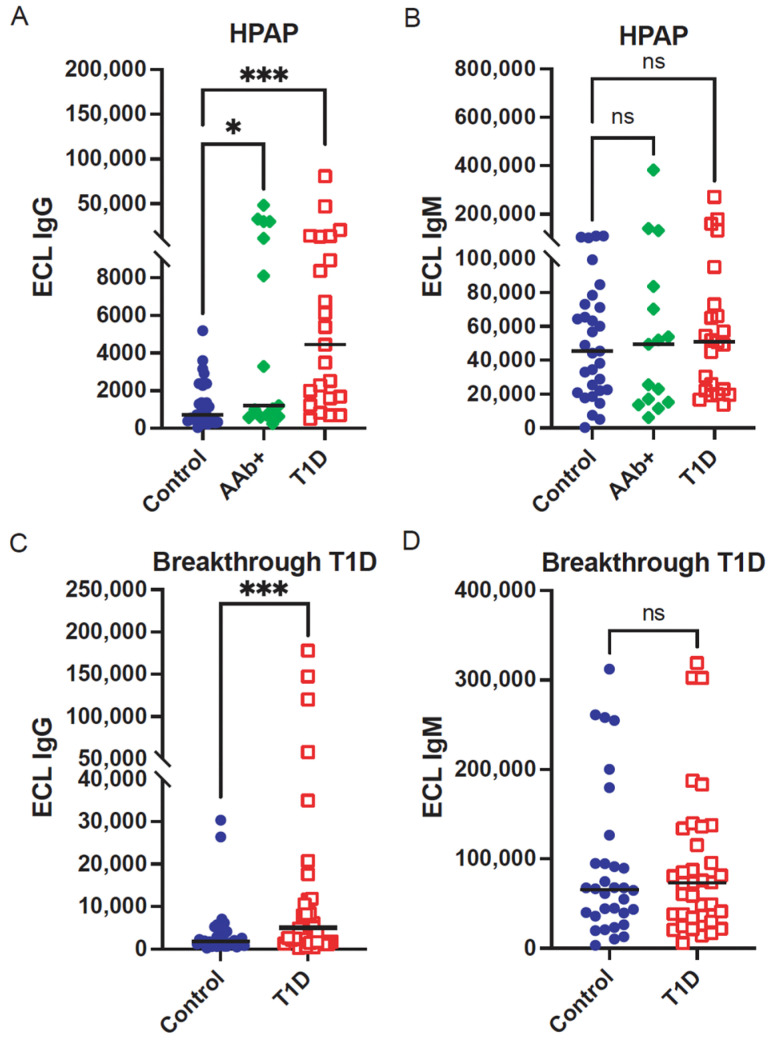
IA2-specific antibody levels by cohort and disease. Blue circles are controls; green diamonds are autoantibody positive but no evidence of disease (AAb+) and red squares are type 1 diabetes (T1D). IgG IA2 levels in HPAP donors (panel (**A**)) and Breakthrough T1D donors (panel (**C**)). IgM IA2 levels in HPAP donors (panel (**B**)) and Breakthrough T1D individuals (panel (**D**)). The Kruskal–Wallis test (Panels (**A**,**B**), two-tailed) and the Mann–Whitney test (Panels (**C**,**D**), two-tailed) were used to evaluate differences between groups. ns, *p* > 0.05, * *p* < 0.05, *** *p* < 0.001. HPAP = Human Pancreas Analysis Program. ECL = electrochemiluminescence.

IgM and IgG anti-IA2 antibodies were measured by ECL on plasma samples from HPAP donors and Breakthrough T1D individuals (Supplementary Figure S4). Compared to the controls, both the HPAP AAb+ and T1D groups showed elevated levels of IA2-IgG (Figure 1A, *p* = 0.041, 0.00026, respectively). The calculated effect size (Є^2^) was 0.28 (H = 19.12), indicating a large difference between the groups. Similarly, IA2 IgG was significantly higher in T1D children than their non-T1D siblings (controls) in the Breakthrough T1D cohort (*p* = 0.00064, Figure 1C). The Rank–Biserial Correlation (r_s_ = 0.40) indicates a substantial IgG effect size. Of note, a few of the sibling controls also exhibited elevated anti-IA2 IgG reactivity. In contrast to IgG autoantibodies, there was no significant difference in IgM anti-IA2 antibodies between the controls, AAb+, and T1D donors in the HPAP cohort (Figure 1B, *p* > 0.05). The calculated IgM effect size (Є^2^) was 0.0011 (H = 0.08), a small effect size. Similarly, there was no significant difference in IgM anti-IA2 antibodies between the controls and T1D donors in the Breakthrough T1D cohort (Figure 1D, *p* > 0.05). The Rank–Biserial Correlation (r_s_ = 0.05) indicates a negligible IgM effect size.

**Figure 2:**
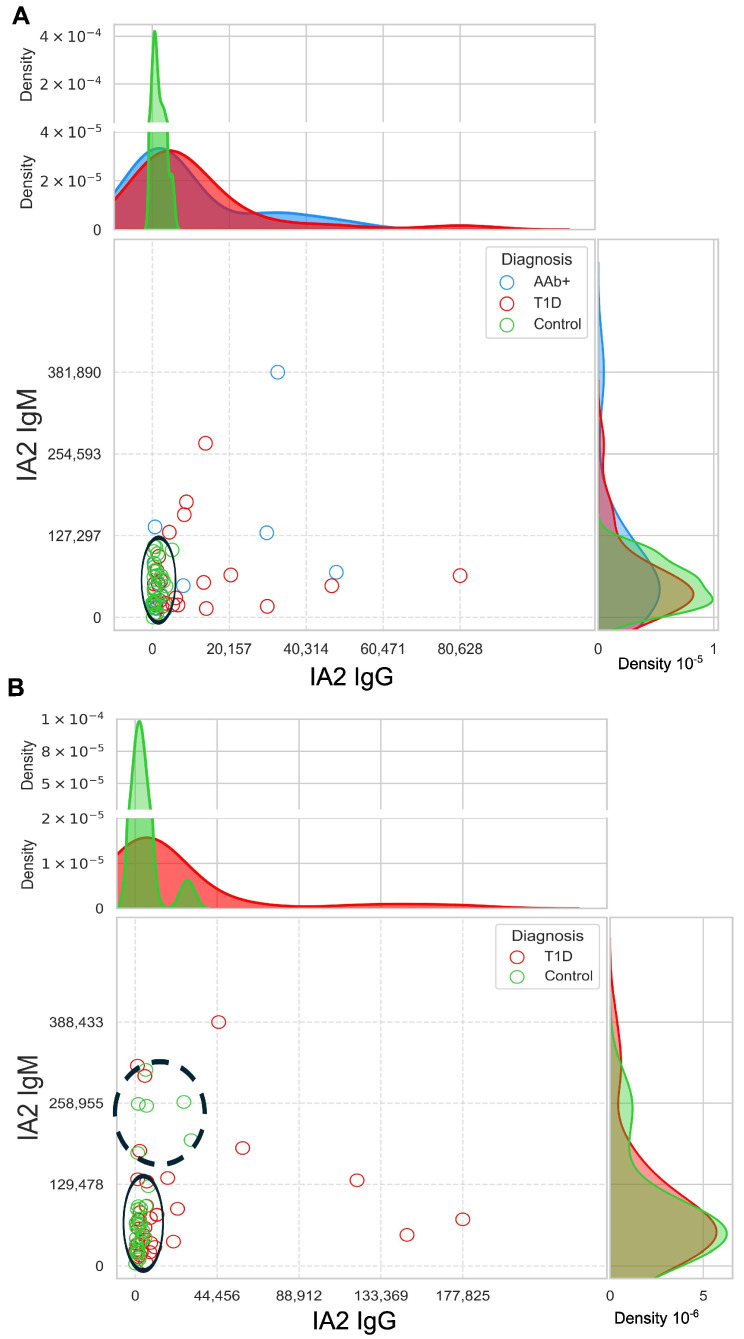
Distribution of IA2-specific IgG and IgM levels in plasma samples from control subjects (green), individuals with positive autoantibodies (AAb+, blue), and T1D (pink) is shown for the HPAP cohort (panel (**A**)) and the Breakthrough T1D cohort (panel (**B**)). Clusters representing low IgM levels (panels (**A**,**B**) lower left, solid black circles) and relatively high IgM levels (panel (**B**), upper left, dashed black circle) among controls are highlighted. Human Pancreas Analysis Program (HPAP); Type 1 diabetes (T1D).

When IgM and IgG antibody levels were compared within individuals and across subject groups, distinct patterns emerged (Figure 2). Some individuals exhibited a predominance of IgG anti-IA2 antibodies, others had mostly IgM antibodies, and still others had elevated levels of both IgG and IgM. Among non-diabetic controls, IgG levels remained within a relatively low, narrow range in both the HPAP (Figure 2A) and Breakthrough T1D cohorts (Figure 2B), whereas IgM levels showed greater variability. Among HPAP T1D and HPAP AAb+ individuals, the distributions of IgG autoantibodies are broad and overlap (Figure 2A). Some control individuals in the Breakthrough T1D cohort, all of whom were first-degree relatives of T1D patients, exhibited significantly elevated IgM reactivity (Figure 2B), which is of interest because the Breakthrough T1D cohort consisted of children, who tend to have higher IgM levels than adults.

**Figure 3:**
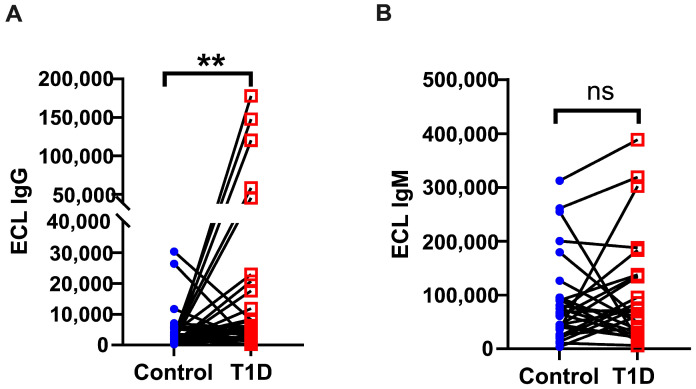
Comparison of IA2-specific IgG (**A**) and IgM (**B**) levels between type 1 diabetes patients (T1D, red squares) and their siblings (Control, blue dots) in the Breakthrough T1D Cohort. Differences between Control and T1D were assessed using a two-tailed paired *t*-test and the Wilcoxon signed-rank test with similar results (ns, *p* > 0.05; ** *p* < 0.01), see text. ECL = electrochemiluminescence.

Next, we wondered if autoantibody levels were correlated within kindreds among the Breakthrough T1D study participants. A comparison of autoreactivity between patients with T1D and their paired first-degree relatives revealed that the T1D group had significantly higher levels of IA2 IgG (*p* < 0.01, Figure 3A), using a paired test (Wilcoxon signed-rank test). The Rank–Biserial Correlation (r_s_ = 0.39) indicates a medium to large effect size. In contrast, IgM levels were not significantly different between the two groups (*p* > 0.05, r_s_ = 0.13) (Figure 3B). Using an unpaired test (Mann–Whitney) as a more stringent evaluation of significance in heterogeneous (rather than fully demographically matched or twin) individuals, we further confirmed that there is a significant difference between T1D and non-T1D siblings in IA2 IgG (*p* = 0.0043), but not in IA2 IgM (*p* = 0.8101).

**Figure 4:**
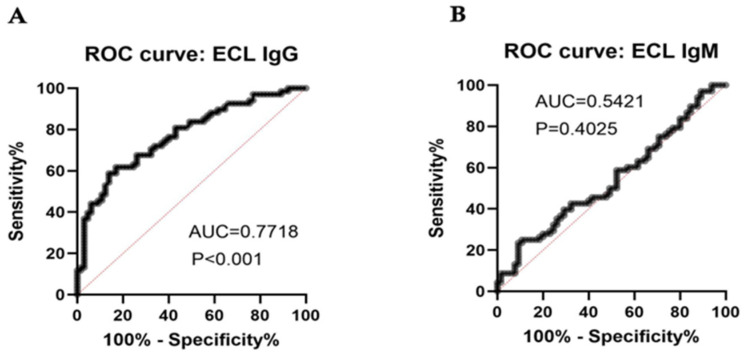
Receiver Operating Characteristic analysis (ROC) of ECL IgG and IgM assays. ROC curves comparing the T1D diagnostic accuracy of (**A**) ECL IgG and (**B**) ECL IgM assays. The black stepped lines represent the trade-off between sensitivity and 100% specificity (false positive rate) across all possible cut-off values, while the diagonal red lines indicate the performance of a random classifier (AUC = 0.50). The ECL IgG assay demonstrated a statistically significant diagnostic value with an Area Under the Curve (AUC) of 0.7718 (*p* < 0.001), while the ECL IgM assay showed poor diagnostic performance with an AUC of 0.5421 (*p* = 0.4025).

To assess the diagnostic value of the ELC IgG and IgM assays, we conducted a Receiver Operating Characteristic (ROC) analysis (Figure 4). The ECL IgG assay demonstrated a statistically significant diagnostic capability with an Area Under the Curve (AUC) of 0.77 (95% CI [0.65, 0.89], *p* < 0.001). IgG AUC performance varied by cohort: 0.84 (95% CI [0.73–0.94]) for HPAP versus 0.71 (95% CI [0.61–0.83]) for Breakthrough T1D. In contrast, the ECL IgM assay showed poor diagnostic performance with an AUC of 0.5421, which did not reach statistical significance (*p* = 0.4025).

**Figure 5:**
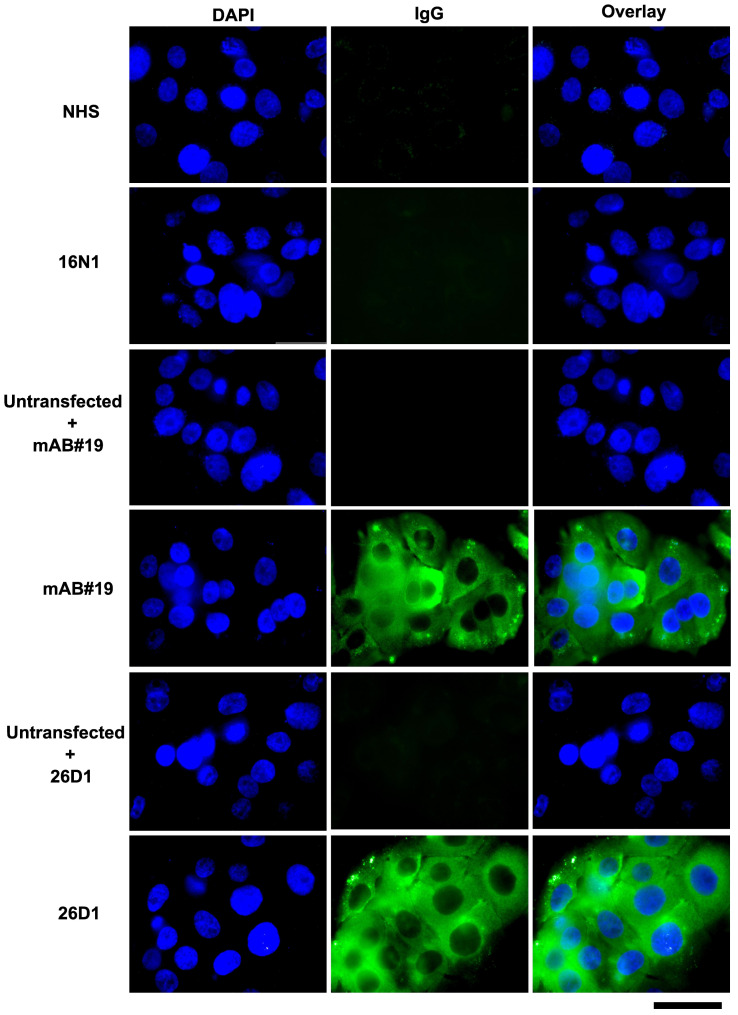
Patient serum autoantibodies bind to intracellular IA2. Immunofluorescence analysis of HaCat (transformed keratinocyte cell line) cells overexpressing the IA2 C-terminus. Cells were incubated with normal healthy serum (NHS, negative control), patient serum (16N1—healthy sibling control, 26D1—T1D), or an anti-IA2 monoclonal antibody (mAb#19, positive control). IgG binding was detected with an AF488-conjugated secondary antibody (Green). Nuclei were counterstained with DAPI (Blue). Scale bar, 100 µm.

To further validate the specificity of our assay for detecting IA2 IgG autoantibodies, we overexpressed the C-terminal portion of IA-2 in a mammalian expression system and compared serum reactivity in transfected vs. untransfected cells (Figure 5). Representative fields with clustered cells showing relatively uniform IA-2 expression were selected to facilitate better image comparison. This cell-based assay performed as expected: negative control sera showed no staining, and a monoclonal antibody that bound IA2 showed positive staining. Of note, a Breakthrough T1D serum sample with previously confirmed IA-2 autoantibodies exhibited specific IgG binding to IA2-expressing cells and no binding to the untransfected control cells (Figure 5).

### 3.3. Correlation of IA2 Reactive IgG with Clinical and Other Autoantibody Serologies

Using the available clinical information in the HPAP cohort, we performed an association analysis between IA2 autoreactivity (IgG and IgM) and T1D diagnosis, age, C-peptide levels, sex, body mass index (BMI), and hemoglobin A1C (HbA1c) using Kendall’s Tau test (Table 2 and Supplementary Figure S5). For HPAP donors, IA2 IgG correlated with glycemic control (HbA1c, *p* = 0.0009), and negatively with C-peptide (*p* = −0.0006) and body mass index (BMI) (*p* = −0.0015, Table 2 and Supplementary Figure S5). In contrast, IA2 IgM was not significantly linked with T1D disease diagnosis or metabolic parameters (Table 2 and Supplementary Figure S5).

We also conducted a correlation analysis using clinical serology results obtained on individuals in both cohorts (Supplementary Figure S6). As expected, our IgG IA2 results were highly correlated with IA2 autoreactivity from the reference laboratory in both cohorts (Supplementary Figure S6, *p* = 0.0005 and 0.0004, respectively). Additionally, IgG IA2 and IgM IA2 had no significant association with GADA and IAA in both cohorts. However, IgG was correlated with antibodies to ZnT8A (*p* = 0.0011) in the Breakthrough T1D group. IA2 IgG correlated significantly with T1D diagnosis in both HPAP (*n* = 69) and Breakthrough T1D (*n* = 65) (*p* = 0.0002 and *p* = 0.0005, respectively), whereas IgM IA2 levels did not correlate with T1D diagnosis (Supplementary Figure S6).

Given the significant association between IgG autoreactivities from our analysis and those from the reference laboratory RBA, we explored the nature of the correlation across the measurement dynamic range. If our method phenocopied the RBA, then it would not necessarily provide new information. If, on the other hand, our method correlated with the RBA, but not linearly, then its relationship with the RBA would be more complex and warrant further study. As a surrogate for the RBA, we used the ECL bridging assay, which correlates linearly with the RBA. Using a linear correlation test, we observed no significant linear correlation between IA2 IgG levels and the bridging assay IA2 autoantibody index, with R^2^ values of 0.1926 and 0.5376 in the HPAP and Breakthrough T1D cohorts, respectively (Supplementary Figure S7A,C, *p* > 0.05). We further observed that some individuals with T1D displayed high IA2 IgG antibody levels but a low IA2 autoreactivity index in the bridging assay, and vice versa. These results indicate that the bridging assay (and, by extension, the RBA) and the IgG ECL detect distinct, non-redundant features of the autoantibody response to IA2. Unsurprisingly, there was also no significant linear correlation between the IgM IA2 ECL and the IA2 bridging assay in either cohort (Supplementary Figure S7B,D). Given the inter-individual variability in the relative amounts of IgM and IgG IA2 antibodies, we wondered if high levels of IgM antibodies might interfere with the detection of IgG antibodies and account for a low linear correlation between the bridging assay and the isotype-specific assays.

### 3.4. Negative Effects of IgM on IA2-Specific IgG Binding

**Figure 6:**
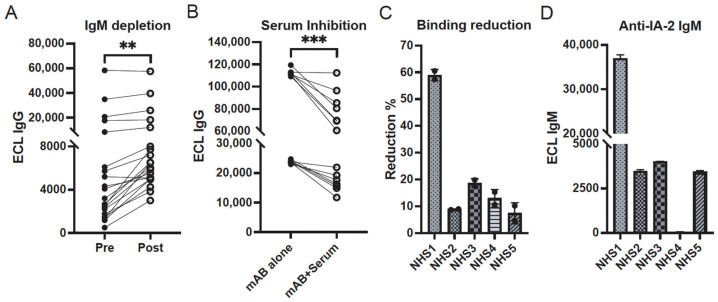
Effect of IgM on IgG binding. (**A**) IA2-specific IgG levels increase post-IgM depletion (post) in some individuals with T1D. ** *p* = 0.006 (two-tailed Wilcoxon signed-rank test). (**B**) Sera with high IA2-specific IgM but low IgG levels significantly inhibited monoclonal anti-IA2 antibody (mAb16 and mAb19) IgG binding to IA2. *** *p* < 0.001 (two-tailed Wilcoxon signed-rank test). (**C**) IgM affinity-purified from the normal human sera (NHS1) reduced mAb binding to IA2 (mAb#19) up to approximately 60%. (**D**) NHS1, but not the other normal donor sera, had relatively high IgM autoreactivity against IA2. NHS = normal human serum; mAb = Monoclonal antibody. ECL = electrochemiluminescence.

To evaluate the effect of IgM on IA2-specific IgG, we depleted total IgM from selected HPAP and Breakthrough T1D plasma samples (that exhibited IA2 reactivity) with an anti-IgM antibody and magnetic beads (see Methods). We then compared IA2 IgG binding levels in untreated vs. IgM-depleted samples using the IgG IA2 ECL assay. The results showed a significant increase in IA2-specific IgG binding after IgM depletion, which was more pronounced in samples with lower initial levels of IgG anti-IA2 autoantibodies (Figure 6A). Furthermore, plasma with high IA2-specific IgM but low IgG levels significantly inhibited monoclonal antibody binding reactivity to IA2 (Figure 6B). Finally, one of five purified IgM preparations from normal human plasma substantially inhibited a monoclonal antibody (HPAP#19) from binding to IA2, although all samples caused a detectable reduction in reactivity (Figure 6C). Interestingly, the plasma sample with the highest blocking activity, NHS1, had a particularly high level of IgM reactivity to IA2 (Figure 6D). Taken together, these results suggest that autoreactive IgM in T1D plasma and in normal plasma can block the binding of IA2-reactive IgG in vitro.

## 4. Discussion

Early and accurate detection of autoantibodies is critical for prompt diagnosis, timely intervention, improved patient outcomes, and a deeper understanding of T1D progression [7,35]. Clinical labs often perform ELISA tests to evaluate serum autoantibodies in individuals at risk for T1D. While the ELISA can detect IgG autoantibodies specific to IA2, we show that its sensitivity and dynamic range are lower than those of ECL. These findings are in line with prior work on these assays using different analytes, such as botulinum toxin [36]. Bridging assays are widely considered the gold standard serologic method for T1D autoantibody evaluation, yet there is substantial discordance between bridging assays and other methods [37]. The ECL bridging assay, which detects total immunoglobulin across all isotypes, yields results that are not linearly correlated with those of the IgG ECL assay. These discrepancies underscore the complexity of T1D serology. In this work, we provide a potential explanation for some of these discrepancies: antibodies of IgM vs. IgG isotypes are detected with different sensitivities; IgG is more highly correlated with T1D; and IgM influences IgG binding in vitro.

The IgM- and IgG-specific ECL assays described herein have advantages over ELISAs and the RBA. First, they only require 1–2 µL of plasma or serum, compared to the 15–41 µL typically needed for RBA or conventional ECL bridging assays [38]. Another advantage of these isotype-specific assays is that they do not use acid treatment of samples, in contrast to the ECL bridging assay [30]. Acid treatment can reduce antibody binding and functional activity, exerting a more significant inhibitory effect on IgM than on IgG [39]. Differences in incubation times and pH may influence the relative abundance of functional IgM vs. IgG in acid-treated samples. Furthermore, because IgM can inhibit IgG binding, as we show here for anti-IA2 antibodies, variations in the relative amounts of IgM and IgG create additional interpretational complexity when these isotypes are combined into a single measurement. In addition to comparing our assays to an RBA and an ECL bridging assay, we further validated the specificity of our assay with a transfected cell line and an anti-IA2 mAb. In the cell line analysis, we showed that serum and an IA2 mAb—which were positive in the ECL assay—bound to cells transfected with the antigen but not to untransfected cells.

With this refined isotype-specific ECL approach, we found that anti-IA2 IgG levels were significantly higher in T1D patients than in controls. While non-diabetic individuals generally showed low and clustered anti-IA2 IgG levels, T1D patients displayed a much wider range of autoantibody levels, with some individuals showing markedly elevated levels. In the HPAP cohort, AAb+ individuals had higher and more variable IgG levels than controls, suggesting they may be in early stages of β-cell autoimmunity and at increased risk of developing T1D [22]. Further, IA2-specific IgG levels were strongly associated with T1D diagnosis and correlated with lower C-peptide levels, higher HbA1c, and lower BMI.

In contrast to IgG, IA2-specific IgM antibody levels did not differ significantly between control and T1D groups in either the HPAP or the Breakthrough T1D cohort. This observation suggests that IgG autoantibodies, rather than IgM, are more relevant for T1D diagnosis. Supporting this possibility, previous studies have shown that IgG insulin autoantibodies are associated with islet cell antibodies (i.e., IgGs bound to islet cells detected by immunofluorescence) in first-degree relatives. In contrast, IgM insulin autoantibodies were more common in individuals without islet cell antibodies [40]. However, we observed some first-degree relatives in the Breakthrough T1D cohort with unusually high IgM levels. In healthy and pre-diabetic individuals, early autoantibody responses are likely dominated by IgM, with a later switch to IgG [41]. Hence, high IgM levels in a subset of AAb+ individuals in the HPAP group and susceptible first-degree relatives in the Breakthrough T1D group could portend a higher risk of disease progression [11,22], but a direct demonstration of this claim would require further studies in longitudinally collected samples. Genetic alterations within the immunoglobulin loci may also contribute to enhanced autoantibody formation and disease progression. One study linked the IGH locus SNP (rs1950942) to both T1D risk and IgG autoantibody reactivity [42], and this same locus is associated with anti-GAD65 IgM autoreactivity in both T1D patients and unaffected individuals.

ROC analysis revealed a combined AUC of 0.77, indicating that IA2 IgG holds significant potential diagnostic value (*p* < 0.001). The moderate nature of this value likely reflects the clinical variability among donors, particularly within the Breakthrough T1D cohort. The Breakthrough T1D cohort includes high-risk controls—unaffected siblings from T1D families—who may possess subclinical autoantibody profiles that confound group separation. This notion is supported by subgroup analysis that demonstrates a higher IgG AUC of 0.84 (95% CI [0.73–0.94]) within the HPAP cohort, compared to 0.71 (95% CI [0.61–0.83]) in the Breakthrough T1D cohort. One intriguing finding was that both T1D patients and controls had variable ratios of IgM and IgG IA2 autoantibodies. Hence, individuals could be categorized as having either IgG-dominant or IgM-dominant autoantibody responses. IgM dominance may reflect an early, potentially reversible stage of autoimmunity, whereas IgG autoantibodies, particularly those targeting multiple β-cell antigens, are strongly associated with T1D development [22]. Separate detection of IgM and IgG autoantibodies may help evaluate the origin of autoimmune responses in individuals receiving immunomodulatory therapies. For example, if patients receive B-cell depletion therapy and experience recurrent autoimmunity, IgM autoantibodies may appear as B cells return to the circulation. Conversely, if patients exhibit persistent autoimmunity, IgG autoantibodies may remain. Secreted IgG antibodies have a longer half-life [43] and tend to be more widely distributed in the body than IgM, with FcRn-mediated protection from degradation and transport across cell barriers [44]. FcRn blockade has emerged as a significant immunomodulatory therapy for autoimmune disease, in large part due to its selective effects on IgG [45]. Here, we show that in vitro, IgM can reduce the detection of IgG binding. We observed that depletion of IgM antibodies from plasma significantly enhanced IgG binding. Conversely, IgM-dominant anti-IA2 sera inhibited monoclonal IgG binding to IA2. These studies suggest that IgM autoantibodies could be nonpathogenic [46] and might interfere with pathogenic IgG autoantibodies. Supporting this concept, we found that purified IgM from normal donor plasma significantly inhibited monoclonal IgG binding to IA2. This inhibitory effect was confirmed using ECL assays, where high levels of IA2-specific IgM were associated with more effective competition for IA2 binding. These in vitro results indicate that IgM and IgG should both be measured, given the effects of one on the detection of the other.

Beyond our findings, which are limited to an in vitro analysis of antibodies, there is growing evidence that autoreactive IgM may play a protective role in vivo by counteracting excessive IgG-driven autoimmunity. Studies in non-obese diabetic (NOD) mice have shown that polyclonal IgM, whether from mice or humans, can protect against diabetes onset [46,47]. Similarly, high-affinity IgM induced by insulin immunization can prevent IgG-driven diabetes in mouse models [48]. In healthy individuals, natural IgM autoantibodies make up 70–80% of circulating IgM [49,50] and are thought to play a key role in immune regulation [51]. These polyreactive IgM antibodies may help maintain immune self-tolerance, preventing excessive immune responses by shielding self-antigens from B cells or by facilitating autoantigen clearance and reducing harmful immune activation. Additionally, polyclonal IgMs from healthy human donors reversed newly developed diabetes by expanding regulatory T-cells (Treg) [46]. IgM may also play a role in regulating IgG stability, independent of competitive binding to antigen. Rheumatoid factors (RF) are primarily IgM antibodies that target the IgG constant region, influencing IgG turnover [52], with low-affinity RF accelerating IgG clearance. Hence, while RF is commonly associated with autoimmunity [53], it could also promote immune complex clearance and exert anti-inflammatory effects.

There are some limitations to this study. Although we have applied our assays to two independent T1D cohorts, the relatively small sample sizes and incomplete clinical data for some subjects limit the generalizability of our findings. Samples from larger, longitudinal studies will be critical for tracking changes in autoreactive IgG and IgM over time and for better defining their association with T1D progression. Additionally, establishing clinically validated thresholds for IgG and IgM positivity will be essential for improving the diagnostic accuracy of these assays.

## 5. Conclusions

Our findings identified distinct, non-redundant contributions of IgG and IgM anti-IA2 antibodies in T1D serology. We developed optimized ECL assays that enabled sensitive, isotype-specific detection of IA2 autoantibodies, requiring minimal sample volume and eliminating the need for acid treatment. Using these assays, we demonstrate that IA2-specific IgG levels in blood plasma are strongly associated with T1D diagnosis. In contrast, IA2-specific IgM in plasma did not differ significantly between T1D and non-T1D individuals. Our results also indicate that IgM can modulate IgG binding in vitro. Separate detection of IgM and IgG autoantibodies could enhance risk stratification, diagnostic accuracy, and improve strategies for early or targeted intervention in T1D.

## Figures and Tables

**Table 1:** Summary of the HPAP and the Breakthrough T1D study cohorts.

**HPAP**	**Number**	**Age (Mean, Year)**	**Age Range (Year)**	**Sex (M)**	**Sex (F)**	
Control	30	25.7	1 to 65	16	14	
AAb+	14	20.4	3 to 30	12	2	
T1D	25	22.4	10 to 42	13	12	
Breakthrough T1D *	**Number**	**Age (Mean, Year)**	**Age Range (Year)**	**Sex (M)**	**Sex (F)**	**Sex** **(unknown)**
Control	33	12.5	6.3 to 20.4	17	10	6
T1D	32	13.5	7.3 to 19.9	18	11	3

This table summarizes the distribution of study participants across the Human Pancreas Analysis Program (HPAP) and Breakthrough T1D cohorts. Participants are stratified by disease status: Control, Autoantibody Positive (AAb+), and Type 1 Diabetes (T1D). Metrics provided include total sample size (number of individuals), mean age, age range, and sex distribution (Male = M, Female = F, or Unknown). * NOTE: For the Breakthrough T1D cohort, age data are only available and shown for *n* = 27 control and *n* = 30 T1D individuals.

**Table 2:** Correlation analysis between IA2 IgM and IgG reactivity and clinical features in HPAP donors.

Group	IA2 IgG	IA2 IgM
HPAP T1D	0.0017 **	0.6537
C-peptide	0.0006 ***	0.1546
Age	0.2101	0.6715
Sex/male	1	0.2969
BMI	0.0015 **	0.0535
Hba1c	0.0009 ***	0.4588

Note: The statistical significance (*p*-value) of the correlation is listed in the table. IgG, but not IgM, is significantly associated with T1D diagnosis (risk), C-peptide levels (negatively), Body Mass Index (BMI, negatively), and HbA1c levels with significance levels of ** *p* < 0.01 and *** *p* < 0.001 (two-tailed, Kendall’s Tau test).

## Data Availability

Data presented in this study are available in the Supplementary Figures and Tables. Further inquiries can be directed to Dr. Luning Prak.

## Supplementary Materials

The following supporting information can be downloaded at https://www.mdpi.com/article/10.3390/biom16040500/s1. Figure S1: Electrochemiluminescence assay platforms used in this study; Figure S2: Comparison of the ECL bridging assay to the IA2 antibody index; Figure S3: ECL is more sensitive than ELISA; Figure S4: Measurement of IA2-specific IgG and IgM autoreactivity in plasma samples; Figure S5: Correlation analysis between IgM and IgG IA2 reactivity and clinical features in HPAP donors; Figure S6: Correlation between IgM and IgG IA2 reactivity and autoantibody index values for IA2 and other T1D-associated autoantibodies; Figure S7: Correlation of IA2-specific IgM and IgG levels with the total IA2 autoantibody index measured by an ECL bridging assay; Table S1: Donors from the HPAP study cohort; Table S2: Patients from the Breakthrough T1D cohort.

## Abbreviations

The following abbreviations are used in this manuscript:

AAb+	Autoantibody positive
ECL	Electrochemiluminescence
ELISA	Enzyme-linked immunosorbent assay
HPAP	Human Pancreas Analysis Program
IA2	Islet antigen 2
IgG	Immunoglobulin G
IgM	Immunoglobulin M
mAb	Monoclonal antibody
MSD	Meso Scale Discovery
NHS	Normal human serum
RBA	Radiobinding Assay
T1D	Type 1 Diabetes
