# Supplementary material for: Differential Contributions of IgM and IgG Autoantibodies to Serologic IA2 Reactivity in Type 1 Diabetes
Source: Biomolecules. 2026 Mar 26;16(4):500. doi: 10.3390/biom16040500 (PMC13113336; doi:10.3390/biom16040500)
Supplement: Supplementary file 1 [file biomolecules-16-00500-s001.zip › biomolecules-4167283-supplementary.pdf]

Supplementary Materials

Differential Contributions of IgM and IgG Autoantibodies to Serologic IA2 Reactivity in Type 1 Diabetes

Xuming Mao, Jake Konigsberg, Nadia Noorchashm, Wenzhao Meng, James J. Knox, Gregory J. Golden, Jacob T. Hamilton, Tara K. Maxwell, Chengyang Liu, Michael R. Betts, Steven M. Willi, Ali Naji, Patrick Hanley and Eline T. Luning Prak

Figure S1: Electrochemiluminescence assay platforms used in this study.

Figure S2: Comparison of the ECL bridging assay to the IA2 antibody index.

Figure S3: ECL is more sensitive than ELISA.

Figure S4: Measurement of IA2-specific IgG and IgM autoreactivity in plasma samples.

Figure S5: Correlation analysis between IgM and IgG IA2 reactivity and clinical features in HPAP donors.

Figure S6: Correlation between IgM and IgG IA2 reactivity and autoantibody index values for IA2 and other T1D-associated autoantibodies.

Figure S7: Correlation of IA2-specific IgM and IgG levels with the total IA2 autoantibody index measured by an ECL bridging assay.

Table S1: Donors from the HPAP study cohort.

Table S2: Patients from the Breakthrough T1D cohort.

Figure S1

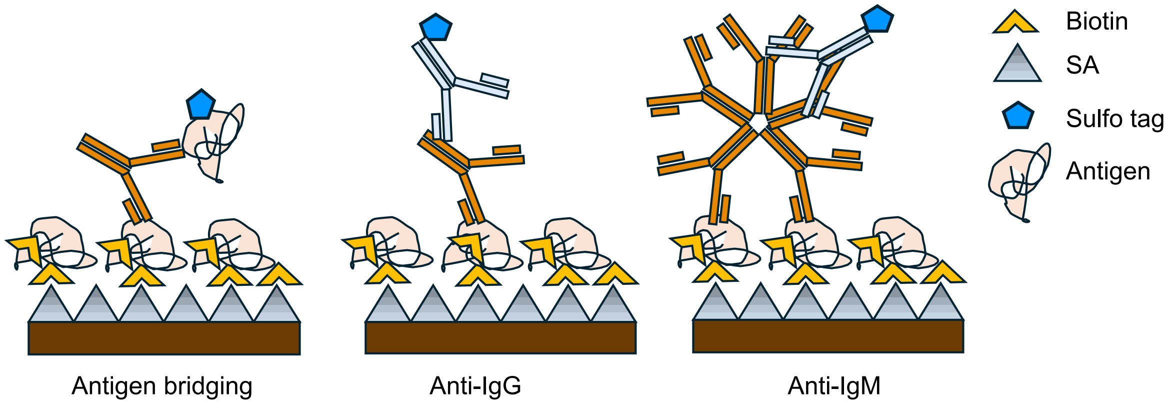

Figure S1. Electrochemiluminescence assay platforms used in this study. In the antigen bridging format, the patient antibody (tan colored immunoglobulin molecule) binds two different labeled forms of the same antigen. A biotinylated form of the antigen is bound to the plate via streptavidin (SA) and a sulfo-tagged version of the same antigen is used to detect the patient antibody. The sulfo tag is conjugated with a chemiluminescent ruthenium trisbipyridal complex that emits light when an antibody-antigen complex is formed and triggered by an electric circuit in the plate. The bridging assayis agnostic to the patient antibody isotype. In the anti-IgG and anti-IgM assays, the biotinylated antigen is bound to the platevia SA, as before, and a secondary sulfo-tagged antibody (blue immunoglobulin) specifically detects either IgG or IgM antibodies from the patient.

Figure S2

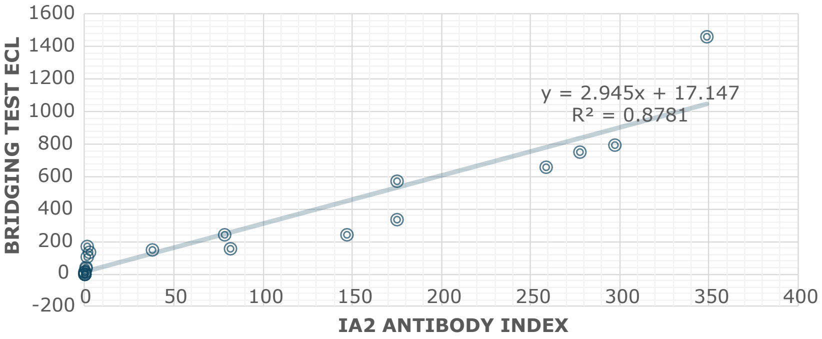

Figure S2. Comparison of the ECL bridging assay to the IA2 antibody index. For the IA2 antibody index from the reference laboratory (see Methods), a mouse monoclonal antibody-125 was used as the assay internal standard positive control, and the result was expressed as an index (index = [Signalsample −SignalNegativeControl] / [SignalPositiveControl − SignalNegativeControl]). For the bridging ECL results from our laboratory, a human monoclonal antibody (mAB#19) was used as internal standard positive control and a normal human serum was used as a negative control. Data points represent 21 samples subjected to the Bridging test. Simple linear regression shows a high linear correlation (R^2=0.8781). The relationship between the two assays was further evaluated using a Spearman correlation analysis (r=0.9741, P<0.0001).

Figure S3

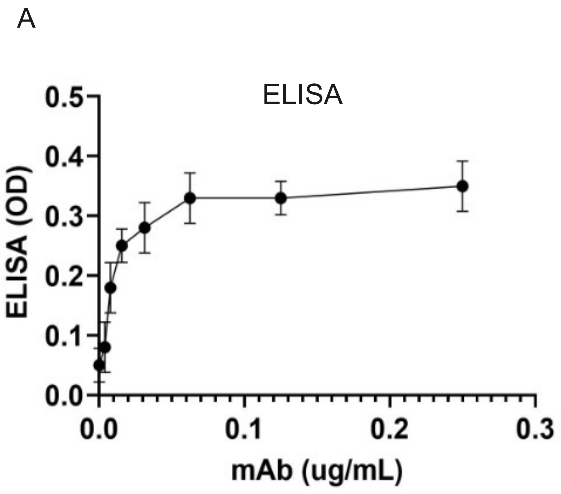

Figure S3. ECL is more sensitive than ELISA. A monoclonal antibody (mAb, designated HPAPmAb#19) directed against the intracellular domain of IA-2 was serially diluted and tested for binding to IA-2 using Enzyme-Linked Immunosorbent Assay (ELISA) and Electrochemiluminescence (ECL). An anti-human IgG labelled with HRP (ELISA) and sulfo-tag (ECL, MSD) was used for detection of binding.

Figure S4

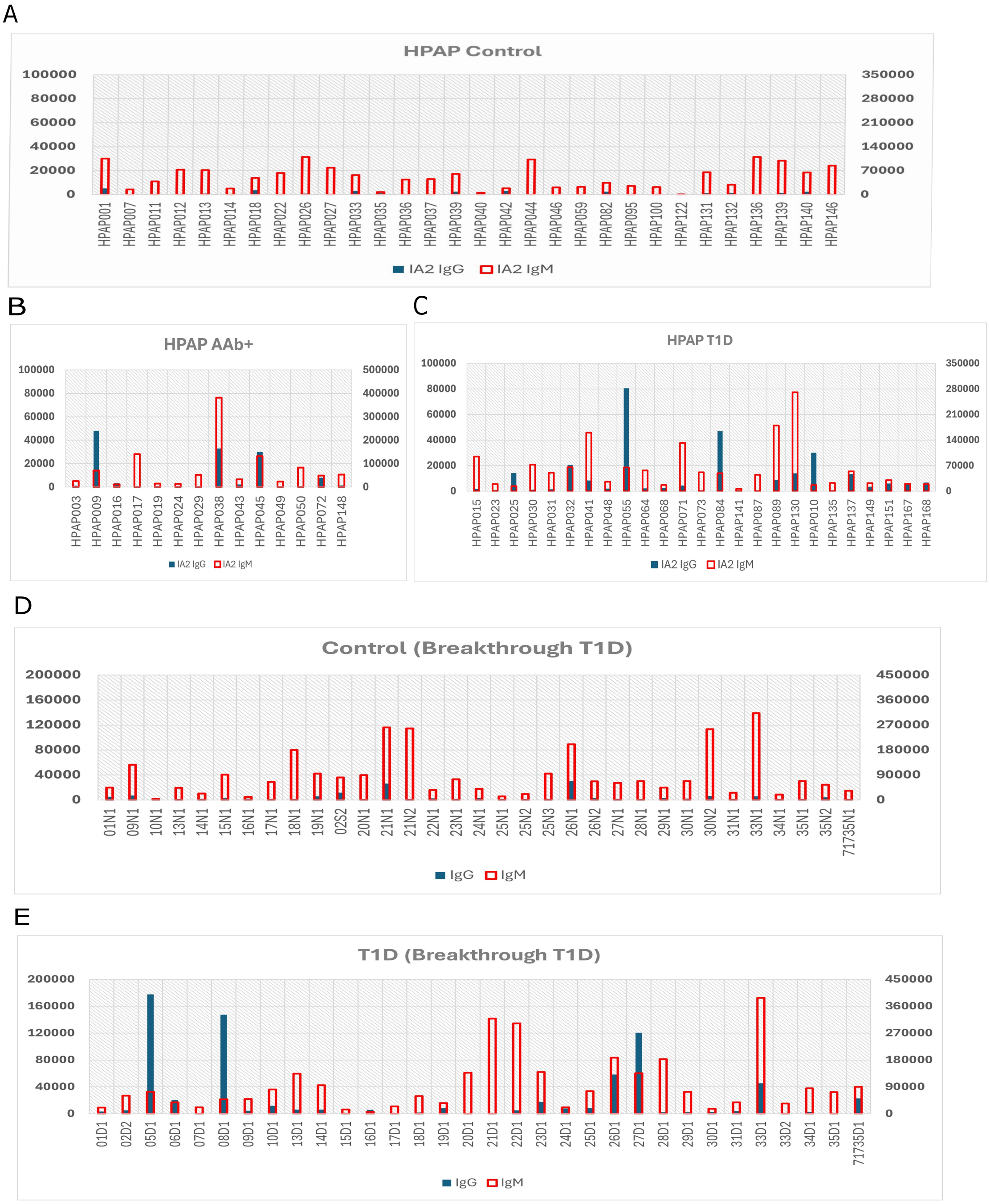

Figure S4: Measurement of IA2-specific IgG and IgM autoreactivity in plasma samples. Measurement of IA2-specific IgG (left y-axis) and IgM autoreactivity (right y-axis) in plasma samples from HPAP control donors (Panel A), HPAP autoantibody-positive (AAb+) donors (Panel B), HPAP donors with T1D (Panel C), Breakthrough T1D control siblings (Panel D) and Breakthrough T1D siblings with T1D using electrochemiluminescence (ECL). HPAP = Human Pancreas Analysis Program; T1D = type 1 diabetes.

Figure S5

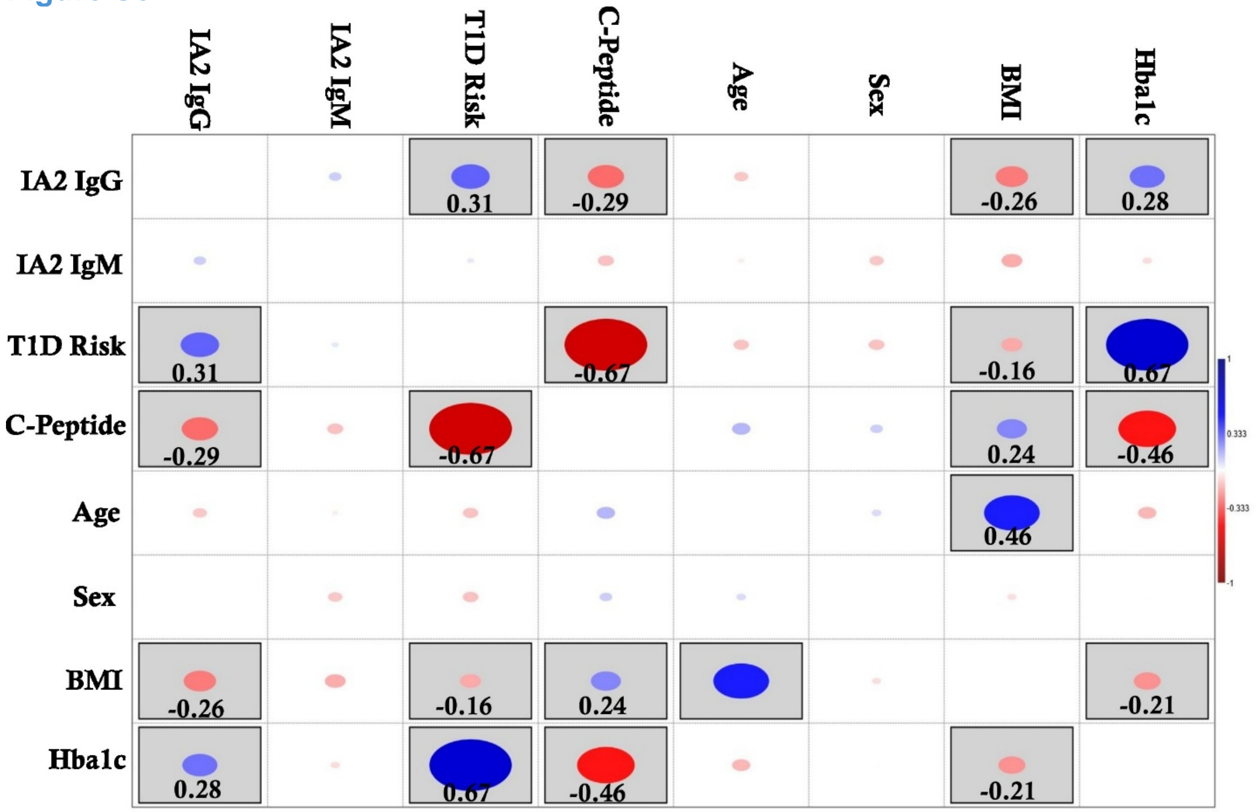

Figure S5: Correlation analysis between IgM and IgG IA2 reactivity and clinical features in HPAP donors. Correlation matrix illustrating the relationship between IA-2 autoantibody isotypes (IgG and IgM) and clinical parameters, including T1D diagnosis, C-peptide levels, age, sex, Body Mass Index (BMI), and glycosylated hemoglobin (HbA1c), within the HPAP cohort. Statistical associations were evaluated using Kendall's Tau (τ) to account for non-parametric distributions. Statistically significant correlations ($P < 0.05$) are highlighted within grey boxed squares, with the numerical τ values indicated. The direction and magnitude of the correlations are visually represented by the color (blue for positive correlation, red for negative correlation).

Figure S6

A

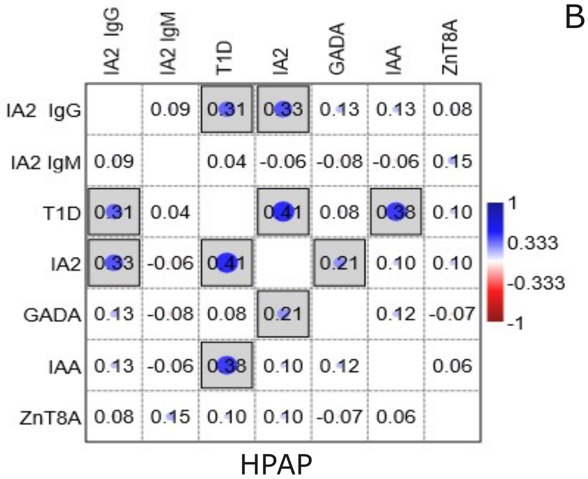

B

Autoantibody	***IA2 IgG***	***IA2 IgM***
**IA2 IgG**		0.2555
**IA2 IgM**	0.2555	
**IA2**	0.0005***	0.5556
**GADA**	0.1501	0.4005
**IAA**	0.1419	0.4603
**ZnT8A**	0.3628	0.0764
**T1D diagnosis**	0.0002**	0.6537

C

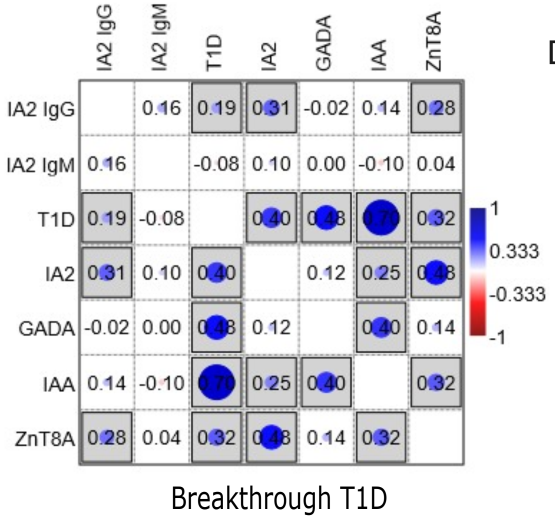

D

Autoantibody	***IA2 IgG***	***IA2 IgM***
**IA2 IgG**		0.0572
**IA2 IgM**	0.0572	
**IA2**	0.0004***	0.2543
**GADA**	0.8307	0.9849
**IAA**	0.1129	0.2317
**ZnT8A**	0.0011**	0.6136
**T1D diagnosis**	0.0005***	0.6361

Figure S6: Correlation between IgM and IgG IA2 reactivity and autoantibody index values for IA2 and other T1D-associated autoantibodies. Correlation between IA2 autoreactivities (IgG and IgM) and the autoantibody index from a reference laboratory—comprising IA2, GAD65, IAA, and ZnT8A—was assessed using the Kendall's Tau test for HPAP (panels A and B) and for Breakthrough T1D (n=65, panels C and D) cohorts. Calculated Tau (τ) values, with significant correlations are indicated in boxed regions (panels A and C, $p < 0.05$, two-tailed). The statistical significance (p-values) of the correlations is detailed in panels B and D, $^{**}p < 0.01$ and $^{***}p < 0.001$ (two-tailed).

Figure S7

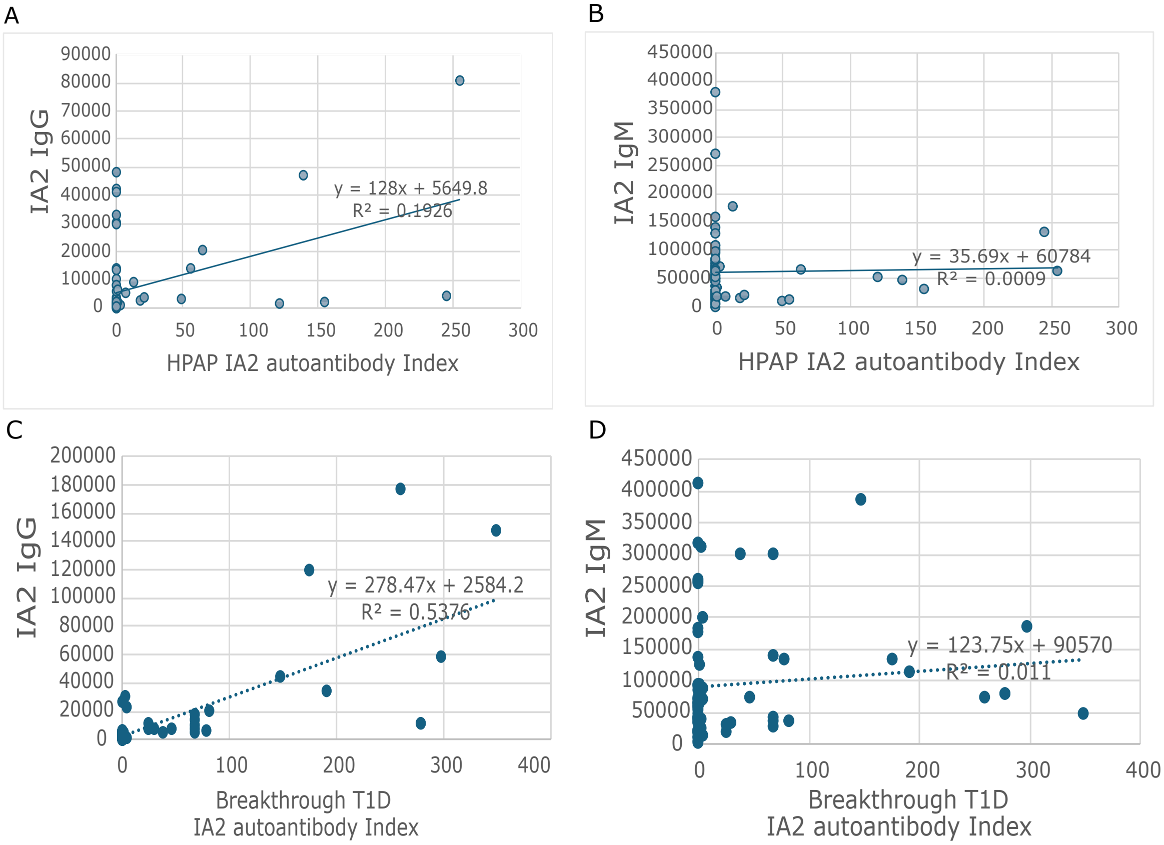

Figure S7: Correlation Analysis of IA2-specific IgM and IgG levels with the total IA2 autoantibody index. IA2-specific IgG levels do not show a significant linear correlation with the total IA2 antibody index (measured by an ECL bridging assay) in the HPAP (panel A) or the Breakthrough T1D (panel C) plasma samples. Similarly, IA2-specific IgM levels do not show a significant linear correlation with the total antibody index in the HPAP (panel B) or the Breakthrough T1D (panel D) plasma samples.

Table S1. Donors from the HPAP study cohort

HPAP Donor	Category	C-Peptide	Age	Sex	BMI	HbA1c	AAb Status
HPAP001	Control	0.43	47	Male	16.4	5.7	
HPAP003	AAb+	9	29	Male	24.5	5.6	GADA Pos
HPAP007	Control	NA	65	Female	42.6	5.9	
HPAP009	AAb+	0.69	3	Male	14.9	NA	GADA Pos
HPAP010	T1D	5.27	42	Female	36.4	6	GADA Pos
HPAP011	Control	1.56	3	Male	17.6	5.5	
HPAP012	Control	4.1	18	Female	29.6	4.5	
HPAP013	Control	4.66	28	Female	41.6	6.3	
HPAP014	Control	6.05	43	Female	30.93	5.7	
HPAP015	T1D	0.03	29	Male	22.03	NA	IAA Pos
HPAP016	AAb+	4.49	30	Male	25.21	5	GADA, IA2, ZnT8 Pos
HPAP017	AAb+	3.71	30	Male	25.8	5.5	GADA Pos
HPAP018	Control	6.4	31	Male	24.47	5.4	
HPAP019	AAb+	8.82	22	Male	29.8	5.2	GADA Pos
HPAP022	Control	9.35	39	Female	34.7	4.7	
HPAP023	T1D	0.02	17	Female	21.35	8.9	IA2 Pos
HPAP024	AAb+	5.6	18	Male	24.59	5.5	GADA Pos
HPAP025	T1D	0.23	30	Male	33.4	8.9	IA2 Pos
HPAP026	Control	0.25	24	Male	20.8	4.9	
HPAP027	Control	7.06	31	Female	32.7	4.4	
HPAP029	AAb+	3.83	23	Male	28.6	5.3	GADA Pos
HPAP030	T1D	0.09	18	Male	21.3	12.4	
HPAP031	T1D	0.02	23	Male	22.64	6.8	IAA Pos
HPAP032	T1D	0.02	10	Female	16.3	9	IA2, IAA Pos
HPAP033	Control	0.27	6	Male	14.85	5.6	
HPAP035	Control	15.9	35	Male	26.91	5.2	
HPAP036	Control	1.12	23	Female	16	5.2	
HPAP037	Control	4.75	35	Female	21.9	5.3	
HPAP038	AAb+	8.29	13	Male	18.34	5.7	GADA Pos
HPAP039	Control	1.88	5	Female	16.3	6.8	
HPAP040	Control	7.01	35	Male	23.98	5.4	
HPAP041	T1D	0.02	24	Female	20.98	7.6	
HPAP042	Control	2.72	1	Male	17.9	5.6	
HPAP043	AAb+	3.19	15	Male	24.1	5.9	IA2, ZnT8 Pos
HPAP044	Control	8.16	3	Female	12	5.3	
HPAP045	AAb+	1.7	27	Female	26.2	5.2	GADA Pos
HPAP046	Control	20.74	19	Male	20.9	5.7	
HPAP048	T1D	0.02	27	Male	25.03	10.2	
HPAP049	AAb+	6.15	29	Male	32.65	5.4	GADA Pos
HPAP050	AAb+	3.79	21	Female	29.13	5.1	GADA Pos
HPAP055	T1D	0.02	24	Male	27.9	10.4	GADA, IA2, IAA, ZnT8 Pos

HPAP059	Control	8.18	35	Male	37.96	5.1	
HPAP064	T1D	0.25	24	Male	16.98	13	ZnT8 Pos
HPAP068	T1D	0.02	20	Female	30.89	13.9	GADA, IA2, IAA Pos
HPAP071	T1D	0.06	12	Female	15.24	9.8	IA2, IAA Pos
HPAP072	AAb+	4.37	19	Male	23.09	5.6	GADA Pos
HPAP073	T1D	0.17	24	Male	25.93	8.2	
HPAP082	Control	2.7	25	Male	23.96	5.6	
HPAP084	T1D	2.2	12	Female	18.48	13.3	GADA, IA2, ZnT8 Pos
HPPA087	T1D	0.02	15	Female	19.2	10.4	IAA Pos
HPAP089	T1D	0.05	27	Female	21.72	10.4	GADA, IA2 Pos
HPAP095	Control	4.13	23	Female	34.32	4.9	
HPAP100	Control	0.67	41	Male	28.83	10.7	
HPAP122	Control	21.16	21	Female	30.5	5.5	
HPAP130	T1D	0.02	32	Female	32.65	8	GADA, IAA Pos
HPAP131	Control	6.53	23	Male	22.5	5.1	
HPAP132	Control	2.88	5	Female	15.72	NA	
HPAP135	T1D	0.25	18	Male	26.35	14.7	
HPAP136	Control	7.14	29	Male	27.24	5.4	
HPAP137	T1D	0.12	23	Male	18.85	16.1	
HPAP139	Control	9.43	22	Male	26.52	5.2	
HPAP140	Control	10.4	29	Female	31.7	4.7	
HPAP141	T1D	9.94	29	Male	31.58	5.7	GADA Pos
HPAP146	Control	9.56	27	Male	28.31	5.5	
HPAP148	AAb+	4.62	7	Male	14.8	5.3	GADA, IA2 Pos
HPAP149	T1D	0.08	11	Male	20	7.8	IA2, IAA Pos
HPAP151	T1D	0.02	31	Male	17.73	14	IAA Pos
HPAP167	T1D	0.07	23	Female	24.28	14	GADA, IA2, IAA Pos
HPAP168	T1D	0.21	14	Female	17.01	14.6	GADA, IAA Pos

Table S1. **Donors from the HPAP study cohort**. Detailed donor-level data for the HPAP cohort, including disease category and key metabolic indicators such as C-peptide levels (ng/mL), glycosylated hemoglobin (HbA1c) (percentage), and Body Mass Index (BMI). Donor age and sex are listed alongside specific autoantibody (AAb) profiles (GADA, IA2, ZnT8, and IAA) where only positive results are recorded. AAb data were generated in a reference lab for the HPAP project (see Methods) and are available on PANC-DB (https://hpap.pmacs.upenn.edu). "NA" denotes values that were not available for specific donors. Type 1 diabetes (T1D); Human Pancreas Analysis Program (HPAP).

Table S2. Patients from the Breakthrough T1D study cohort.

Breakthrough T1D	Category	c-peptide	Age	HbA1c	Sex
1D1	T1D	0.3	17.8	>14	Male
1N1	Control	NA	14.5	NA	Male
2D2	T1D	NA	11.6	11.9	Female
2N (labeled 2S2)	Control	NA	12.5	NA	NA
5D1	T1D	0.9	9.5	>14	NA
6D1	T1D	1.4	17.4	10.6	NA
7D1	T1D	0.5	15.1	>14	Female
8D1	T1D	0.8	13.5	>14	Male
9D1	T1D	0.691	16.2	7.2	Male
9N1	Control	NA	12.8	NA	Male
10D1	T1D	1.4	18.7	8.4	Female
10N1	Control	NA	14.1	NA	Male
13D1	T1D	0.4	11	12.7	Female
13N1	Control	NA	11	NA	Male
14D1	T1D	NA	16.5	11.4	Male
14N1	Control	NA	15.6	NA	Male
15D1	T1D	0.3	10.8	12.3	Male
15N1	Control	NA	13.5	NA	Male
16D1	T1D	NA	8.9	12.9	Male
16N1	Control	NA	14.1	NA	Male
17D1	T1D	NA	16.7	5.1	Female
17N1	Control	NA	20.4	NA	Female
18D1	T1D	NA	19.6	NA	Male
18N1	Control	NA	15.4	NA	Female
19D1	T1D	0.994	11.9	9.44	Male
19N1	Control	NA	13.7	NA	Male
20D1	T1D	0.617	19.9	>14	Female
20N1	Control	NA	18.7	NA	Male
21D1	T1D	0.271	11.5	12.1	Male
21N1	Control	NA	13.1	NA	Female
21N2	Control	NA	9.8	NA	Male
22D1	T1D	0.8	7.3	9.1	Female
22N1	Control	NA	9.2	NA	Female
23D1	T1D	0.5	13.7	>14	Male
23N1	Control	NA	6.4	NA	Male
24D1	T1D	NA	12.1	11.9	Male
24N1	Control	NA	16.9	NA	Male
25D1	T1D	1.1	13.1	9.7	Male
25N1	Control	NA	11.6	NA	Male
25N2	Control	NA	9.1	NA	Female

25N3	Control	NA	9.1	NA	Female
26D1	T1D	0.2	8.4	11.5	Female
26N1	Control	NA	10.8	NA	Female
26N2	Control	NA	6.3	NA	Male
27D1	T1D	0.2	13.3	>14	Male
27N1	Control	NA	12.3	NA	Male
28D1	T1D	NA	11.6	8.9	Male
28N1	Control	NA	9.6	NA	Male
29D1	T1D	NA	9.2	11.94	Female
29N1	Control	NA	7.5	NA	NA
30D1	T1D	0.192	16.1	11.6	Male
30N1	Control	NA	17.9	NA	NA
30N2	Control	NA	NA	NA	Female
31D1	T1D	0.672	14	10.25	Male
31N1	Control	NA	12.4	NA	Female
33D1	T1D	0.186	9.3	>14	Female
33D2	T1D	NA	14	>14	Female
33N1	Control	NA	NA	NA	Female
34D1	T1D	0.354	16.3	9.9	Male
34N1	Control	NA	NA	NA	Female
35D1	T1D	NA	NA	NA	Male
35N1	Control	NA	NA	NA	NA
35N2	Control	NA	NA	NA	NA
71735D1	T1D	NA	NA	NA	NA
71735N1	Control	NA	NA	NA	NA

Table S2. **Patients from the Breakthrough T1D Study Cohort.** Profile of participants in the Breakthrough T1D study, donor IDs (donors with the same prefix number come from the same family), disease classification (T1D vs. Control), and available clinical measurements. Data fields include C-peptide concentration (ng/mL), age at sampling (years), HbA1c (percentage), and sex. Marked "NA" indicates missing or unavailable data points. Type 1 diabetes (T1D).
